# Editorial: Micro and nanoparticles for regenerative medicine

**DOI:** 10.3389/fbioe.2024.1548963

**Published:** 2025-01-13

**Authors:** Aurore Van de Walle, Yang Zhang, Alessandro Polini, Riccardo Di Corato

**Affiliations:** ^1^ Laboratoire Physique des Cellules et Cancer, Institut Curie, Centre national de la recherche scientifique (CNRS), Université PSL, Paris, France; ^2^ School of Dentistry, Shenzhen University Medical School, Shenzhen, China; ^3^ Institute of Nanotechnology (CNR-NANOTEC), National Research Council, Lecce, Italy; ^4^ Institute for Microelectronics and Microsystems (CNR-IMM), National Research Council, Lecce, Italy

**Keywords:** microparticle, nanoparticle (NP), regenerative medicine, tissue engineering, antibacterial activity

## Introduction

To address regenerative medicine challenges comes the need for innovative biomaterials, meticulously tailored to specific functions. Their design can be optimized to support human cell repopulation and regulate phenotype, facilitating tissue regeneration. Alternatively, they can be engineered to combat bacterial growth, as the race against bacterial resistance is becoming pressing. Particles in the micrometer or nanometer range provide original, size-dependent, and tunable physiochemical features. This Research Topic “*Micro- and Nano-Particles for Regenerative Medicine*” reports on creative syntheses, compositions, and applications of these biomaterials.

## Toward eco-friendly syntheses

The synthesis of micro- and nano-particles must be precisely controlled to achieve the desired shape and composition, as these characteristics profoundly influence their applicative efficacy. A large body of literature aims at constantly improving structure/function of these materials by refining synthesis procedures. Within it, a growing field of chemistry focuses on green synthesis methods to provide more sustainable alternatives while maintaining the bioactive efficiency of the particles. For instance, this Research Topic features a study on magnesium oxide (MgO) nanoparticles synthesized using Azadirachta indica (neem) extract (Al-Harbi et al.). The produced MgO nanoparticles exhibit remarkable stability under heat and in biological media, alongside notable antioxidant, anti-inflammatory, and antibacterial properties. Aligned with this search for greener processes and materials, another featured study reviews the development of silk fibroin-based scaffolds for tissue engineering (Ma et al.). Silk, biosynthesized by over 200,000 arthropod species - including the *Bombyx mori* moth, whose silk is the most characterized–offers a rich source of fibroin, a natural biopolymer. Silk fibroin stands out for its mechanical strength, biocompatibility, biodegradability, and structural tunability. It can be used alone or combined with other materials to enhance cell adhesion, proliferation, and differentiation.

## Tissue engineering applications

A focus in regenerative medicine consists in creating tissue alternatives to repair aging or diseased tissues and organs. It generally relies on the use of a scaffold for cell support. These scaffolds must meet precise guidelines to provide appropriate biomechanical and biochemical cues to facilitate cell adhesion and regulate phenotypic expression. Their conception thus requires control over shape, mechanical properties, and surface characteristics. The previously mentioned review on silk fibroin details the numerous possible processing techniques of this biomaterial (i.e., electrospinning, freeze-drying, solvent casting, gas foaming, particulate leaching, 3D printing) making it versatile and relevant for the repair of various tissues, including skin, bone, cartilage, blood vessel, and nerves (Ma et al.). Nano- and micro-structuration of scaffolds is another approach to enhance scaffold features as explored in this Research Topic in a study that showcases the incorporation of graphene oxide to the pure hardystonite (used for bone repair) leading to the obtention of a porous nano- and microstructured hardystonite/reduced graphene oxide composite (Bagherpour et al.). The produced biomaterial demonstrates enhanced osteogenic differentiation and mechanical strength. An additional study focuses on the nano-scale properties of biphasic calcium phosphate (BCP), also employed in bone repair (Su et al.). Granular-shaped BCP nanoparticles were synthesized, and the nanoscale texturing of these materials enhanced biocompatibility and osteogenic features. Importantly, they demonstrated immunomodulatory effects by reducing the expression of pro-inflammatory cytokines in macrophages, even under lipopolysaccharide (LPS)-induced inflammatory conditions. The ability of BCP nanoparticles to restore osteogenesis impaired by inflammation underscores their dual role in promoting bone regeneration and modulating the immune response.

## Bioactivity offered by nanomaterials and therapeutic potential

Nanometer-scaled materials can provide stronger antibacterial features. The silver-copper bimetallic nanoparticles featured in this Research Topic exemplify this strategy (Hao et al.). Silver and copper nanoparticles have superior antibacterial effects compared to their bulk or micron-size counterparts. Furthermore, combining these nanomaterials rather than using them individually amplifies their antibacterial efficacy while minimizing cytotoxicity. These nanoparticles have dynamic interactions in biological environments through metal ions release and reactive oxygen species (ROS) generation, which collectively boost localized antibacterial activity, minimizing systemic side effects (Hao et al.).

Bioinspired MgO NPs also exhibited targeted effects. They show selective cytotoxicity against liver hepatic cancer cells in comparison to human umbilical vein endothelial ones, encouraging their potential in anticancer therapies (Al-Harbi et al.). Additionally, they demonstrate significant antidiabetic activity through the inhibition of key enzymes like α-glucosidase and α-amylase, along with robust anti-inflammatory effects via protein stabilization and cytokine regulation. Antioxidant activity is another critical aspect of bioactive particles. By scavenging free radicals, MgO NPs protect tissues from oxidative stress, a major contributor to inflammation and chronic diseases.

Similarly, BCP nanomaterials have shown remarkable bioactivity. They mitigate inflammation in macrophages and enhance osteogenic processes by increasing alkaline phosphatase activity and upregulating osteogenesis-related genes, such as Runx2 and OPN (Su et al.). Notably, BCP nanoparticles can reverse the detrimental effects of lipopolysaccharide (LPS)-induced inflammatory conditions on osteoblast activity. This dual functionality makes BCP nanoparticles promising candidates for applications in environments where inflammation impedes bone regeneration.

## Future directions

The integration of micro- and nano-particles into regenerative medicine can not only support tissue regeneration but also actively engage in therapeutic processes. While these versatile biomaterials hold large promises, challenges still remain for their widespread adoption in the clinic. Among them, the assessment of long-term biocompatibility requires monitoring biomaterial evolution over years. It is a complex task, yet necessary, as these materials are designed to interact with biological systems over extended periods**.** Another challenge concerns their integration with advanced manufacturing, such as 3D printing, as well as the scalability and reproducibility of their manufacturing. This is necessary to ensure consistent production of high-quality particles for large-scale applications. In parallel to these technical hurdles come regulatory and translational barriers. However, as this field progresses and with sustained efforts to overcome these obstacles, we are optimistic that micro- and nano-particles can pave the way for more effective medical solutions.

